# Identifying Optical Coherence Tomography Markers for Multiple Sclerosis Diagnosis and Management

**DOI:** 10.3390/diagnostics13122077

**Published:** 2023-06-15

**Authors:** Larisa Cujba, Cristina Stan, Ovidiu Samoila, Tudor Drugan, Ancuta Benedec (Cutas), Cristina Nicula

**Affiliations:** 1 Medical Doctoral School, University of Oradea, 410087 Oradea, Romania; 2 Department of Ophthalmology, Faculty of Medicine, “Iuliu Hatieganu” University of Medicine and Pharmacy, 400006 Cluj-Napoca, Romania; 3 Department of Medical Informatics and Biostatistics, “Iuliu Hațieganu” University of Medicine and Pharmacy, 400012 Cluj-Napoca, Romania

**Keywords:** multiple sclerosis, neurodegeneration, clinically isolated syndrome, optical coherence tomography, retinal fiber layers, ganglion cell layer, posterior pole analysis, macular retinal segmentation

## Abstract

Background: Multiple sclerosis (MS) is a common neurological disease affecting the optic nerve, directly or indirectly, through transsynaptic axonal degeneration along the visual pathway. New ophthalmological tools, arguably the most important being optical coherence tomography (OCT), could prove paramount in redefining MS diagnoses and shaping their follow-up protocols, even when the optic nerve is not involved. Methods: A prospective clinical study was conducted. In total, 158 eyes from patients previously diagnosed with relapsing remitting MS (RRMS)—with or without optic neuritis (ON), clinically isolated syndrome (CIS) with or without ON, and healthy controls were included. Each patient underwent an ophthalmologic exam and OCT evaluation for both eyes (a posterior pole analysis (PPA) and the optic nerve head radial circle protocol (ONH-RC)). Results: The macular retinal thickness (the 4 × 4, respectively, 2 × 2 grid) and thickness of the peripapillary retinal nerve fiber layer (pRNFL) were investigated. Various layers of the retina were also compared. Our study observed significant pRNFL thinning in the RRMS eyes compared to the control group, the pRNFL atrophy being more severe in the RRMS-ON eyes than the RRMS-NON eyes. In the ON group, the macular analysis showed statistically significant changes in the RRMS-ON eyes when compared only to the CIS-ON eyes, regarding decreases in the inner plexiform layer (IPL) thickness and inner nuclear layer (INL) on the central 2 × 2 macular grid. The neurodegenerative process affected both the inner retina and pRNFL, with clinical damage appearing for the latter in the following order: CIS-NON, CIS-ON, RRMS-NON, and RRMS-ON. In the presence of optic neuritis, SMRR patients presented an increase in their outer retina thickness compared to CIS patients. Conclusions: To differentiate the MS patients from the CIS patients, in the absence of optic neuritis, OCT Posterior Pole Analysis could be a useful tool when using a central 2 × 2 sectors macular grid. Retinal changes in MS seem to start from the fovea and spread to the posterior pole. Finally, MS could lead to alterations in both the inner and outer retina, along with pRNFL.

## 1. Introduction

Multiple sclerosis (MS) is the most common inflammatory neurological disease of the central nervous system that affects young adults [1,2].

Regarding the pathogenesis of MS, the following processes are involved in the destruction of the myelin sheath: neurodegeneration, neuroinflammation, autoimmune response, excitotoxicity, and gliosis [3].

Considering neurodegeneration, the underlying cause of disability in MS, intensive research has been conducted in order to identify markers able to quantify and track these neuronal changes. The retina offers the opportunity to study the central nervous system, with retinal changes in MS reflecting both focal and global aspects of neurodegeneration and inflammation [4].

The most common subtype of MS is relapsing-remitting MS (RRMS), which comprises 85% of all cases [5].

Clinically isolated syndrome (CIS) is typically the first and earliest clinical manifestation of MS, making patients with this diagnosis vital for research and offering doctors the opportunity to evaluate the earliest signs and quantify the changes in MS [6].

The last revision of the MS diagnostic criteria proposed introducing the optic nerve as the fifth CNS location to quantify the dissemination in time and space of its characteristic lesions, therefore emphasizing the importance of precise evaluations of the optic nerve in refining this diagnostic criteria [7].

Considering The 2017 revisions of the McDonald criteria for the diagnosis of multiple sclerosis: typical CIS-presenting clinical or magnetic resonance imaging (MRI) evidence of dissemination in space of the central nervous system’s (CNS) lesions, along with cerebral spinal fluid (CSF)-specific oligoclonal bands, allow for the diagnosis of multiple sclerosis [7].

Visual dysfunction is common in MS, usually occurring after optic neuritis (ON). Less frequently, however, demyelinating lesions can affect the retro-chiasmatic visual pathway [8].

Many MS lesions are asymptomatic, including optic nerve ones. Their early detection would prevent diagnoses at advanced stages of the disease [9]. Identifying subclinical lesions of the visual pathways has become a significant aspect in the evaluation of newly diagnosed cases of MS [10,11].

Optic neuritis is an inflammatory lesion, frequently involving the retrobulbar portion of the optic nerve. It represents the most well-defined clinically isolated syndrome associated with MS [5]. Optic neuritis is the presenting complaint in 20% of multiple sclerosis patients [12]. Furthermore, up to 70% of patients with MS develop an episode of neuritis during the course of the disease [13].

Optical coherence tomography (OCT) is a fast, inexpensive, reproductible, and noninvasive imaging technique. It uses near-infrared light to generate cross-sections or three-dimensional images of the retina [14]. Imaging biomarkers of the retinal structure are important for recognizing and monitoring the inflammation and neurodegeneration in MS. With the advent of spectral domain OCT (SD-OCT), the supervised automatic segmentation of each individual retinal layer is possible. The essential changes observed in those with MS were in the macular ganglion cell-inner plexiform layer (GCIPL) and peripapillary retinal nerve fiber layer (pRNFL) [15]. These two elements are recommended to be used for diagnoses, monitoring, and research. The inflammatory activity of the disease could also be captured by the changes observed in the inner nuclear layer [15].

Considering the difficulties in diagnosing MS, especially in CIS cases, new ophthalmological tools, arguably the most important being OCT, could prove paramount in redefining MS diagnoses and shaping their follow-up protocols, even when the optic nerve is not involved.

Previous studies have demonstrated the phenomenon of transsynaptic axonal degeneration along the visual pathway in MS. We want to identify the OCT markers that are characteristic of MS patients, as well as their patterns, in order to differentiate MS-ON patients from MS-NON patients and MS patients from CIS patients. In this regard, we want to analyze the utility of a less used macular test grid for quantifying the retinal changes in all the layers of the eye in MS patients, in order to develop better diagnosis and monitoring tools for neurodegeneration. Identifying the subclinical activity of this disease with the use of optical coherence tomography is a secondary aim of the study, in order to facilitate earlier diagnoses and the easier surveillance of the global neurodegenerative process in clinical practice.

## 2. Materials and Methods

### 2.1. Study Design

The present study is analytical, observational, and monocentric. The selection of the participants was based on selective, convenient sampling, with the data collection being performed prospectively. The subjects gave their informed consent to participate in the study. The study respected the principles of the Declaration of Helsinki and the research protocol was approved by the Ethics Committee of the Iuliu Hațieganu University of Medicine and Pharmacy, Cluj-Napoca (No. 227/22.06.2020), and the Research Ethics Committee of the Faculty of Medicine and Pharmacy, University of Oradea (No. CEFMF/02 of 31.10.2022). The patients were enrolled between November 2021 and March 2022.

### 2.2. Participants

A total of 170 eyes were evaluated for the study, 158 were included in the final analysis, and 12 eyes were excluded.

We included 54 eyes of healthy subjects, 64 eyes of relapsing-remitting multiple sclerosis (RRMS) patients with prior optic neuritis (RRMS-ON), 26 eyes from RRMS patients without a history of optic neuritis (RRMS-NON), 6 eyes from clinically isolated syndrome (CIS) patients with prior optic neuritis (CIS-ON), and 8 eyes of CIS patients without a history of optic neuritis (CIS-NON). The ON patients were evaluated at least 3 months after an acute episode.

The MS and CIS patients were recruited from the Neurology Department of the Cluj-Napoca Emergency County Hospital. The healthy age- and sex-matched subjects were selected during the same time from the Ophthalmology Department of the same hospital.

The inclusion criteria for this study were: (1). patients assigned by the Specialty Commission for Multiple Sclerosis with a definite diagnosis of multiple sclerosis (RRMS); (2). patients diagnosed with CIS by the neurological department; (3). patients who presented at the clinic at least 3 months after an optic neuritis episode (documentation required); (4). patients able to understand the instructions provided; and (5). patients who gave their written consent to participate in the study

The exclusion criteria were: (1). patients with other ocular or systemic pathologies whose consequences overlap with the researched pathology; (2). patients without an acceptable SD-OCT evaluation; (3). a history of posterior pole surgery, cranio-cerebral trauma, cerebral vascular accidents, meningitis, encephalitis, or brain tumors; and (4). patients with mental health issues and those without temporal and spatial orientation.

### 2.3. Clinical and Paraclinical Assessment

Each subject was evaluated by the same team of ophthalmologists recording the following data: best-corrected visual acuity (BCVA) (measured with the Snellen scale), anterior and posterior pole biomicroscopy evaluation, aplanotonometry (Goldman, New York, NY, USA), visual field 30-2 (Humphrey Field Analyzer, Carl Zeiss Meditec, Dublin, CA, USA), and OCT measurements (Spectralis, Heidelberg Engineering GmbH, Heidelberg, Germany). ONH-RC and posterior pole analyses were used. Personal data were collected, such as: age, gender, level of education, the clinical form of MS, the ophthalmological onset of the disease, the number of years since receiving the diagnosis, the presence or absence of optic neuritis episodes, the presence or absence of MS-specific treatment upon entering the study, and EDSS score. All the RRMS and CIS patients had undergone a brain MRI with contrast within 3 months prior to entering the study.

### 2.4. The Methodology of OCT Measurements

The OCT measurements were performed on both eyes of each participant by a single technician who used the same Spectral Domain OCT device (Spectralis, Heidelberg Engineering GmbH, Heidelberg, Germany), which has a built-in real-time eye-tracking system that records the movements of eyeballs and generates feedback to the scan mechanism to stabilize the scan position.

**Figure 1:**
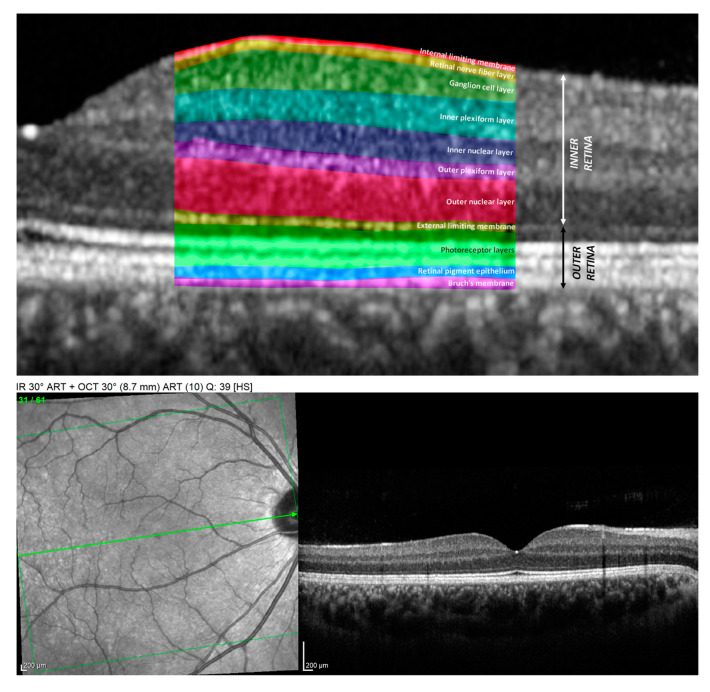
Cross-section images of the macula obtained using SD-OCT. Upper image represents the magnified view of the retinal layers as follows: internal limiting membrane (IML), mRNFL, GCL, IPL, INL, OPL, ONL, external limiting membrane (ELM), photoreceptors layers (PR), RPE, and Bruch membrane (BM). The image bellow: on the left: “En face” OCT scan of the macula (green line showing the level of the scan through the center of the fovea), on the right: cross-section of the macula corresponding to a healthy control.

The scans of both the optic nerve and macular region were recorded using ONH-RC and posterior pole analysis protocols. The global and sector thickness of the pRNFL, as well as the macular thickness (a central grid of 2 × 2 and 4 × 4 central sectors) on the 7 individually targeted layers: the macular retinal nerve fiber layer (mRNFL), ganglion cell layer (GCL), inner plexiform layer (IPL), inner nuclear layer (INL), outer plexiform layer (OPL), outer nuclear layer (ONL), retinal pigment epithelium (RPE), and total macular thickness (TMT), were compared between the groups. Figure 1 summarizes all the retinal layers.

**Figure 2:**
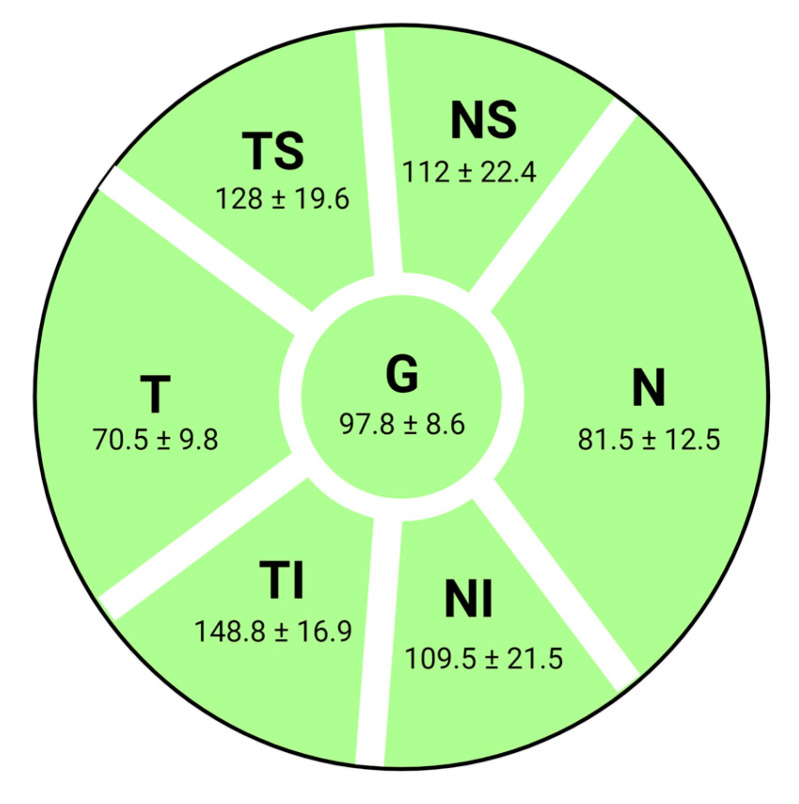
OCT diagram of optic nerve head sectors measurements (right eye) and correspondent RNFL thickness values (µm) from normative database [16]: G—global average, TS—temporal superior, T—temporal, TI—temporal inferior, NI—nasal inferior, N—nasal, and NS—nasal superior.

The pRNFL analysis was performed by scanning a 3.5 mm circumferential area centered on the optic nerve head, resulting in a pie chart (Figure 2) divided into 7 sectors (G—global average, TS—temporal superior, T—temporal, TI—temporal inferior, NI—nasal inferior, N—nasal, and NS—nasal superior) that recorded the pRNFL’s mean thickness value in microns for each quadrant.

**Figure 3:**
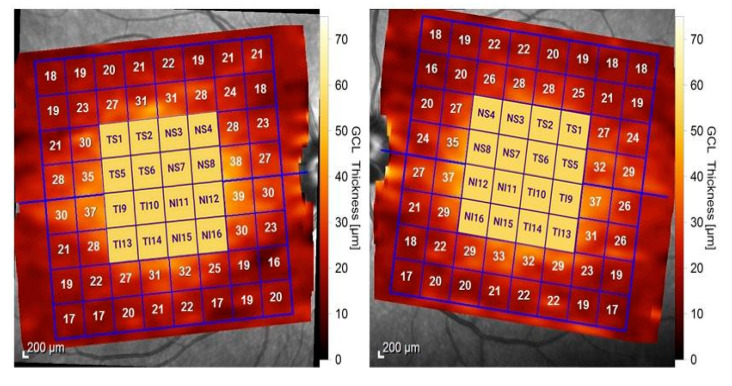
SD-OCT macular scan using posterior pole analysis. We analyzed the 16 central sectors numbered from 1 to 16, divided into nasal sectors (NS3, NS4, NS7, NS8, NI11, NI12, NI15, and NI16) and temporal sectors (TS1, TS2, TS5, TS6,TI9, TI10, TI13, and TI14). Superior sectors were from 1–8 and inferior sectors were from 9–16.

The analysis of the macular retina was carried out using the posterior pole analysis protocol of Heidelberg SD-OCT, which uses the APS (Anatomic Positioning System) system to adjust the acquisitions to the unique axis of each eye (FoBMOC, fovea-to-Bruch’s membrane opening center). This aspect allows for an elimination of the variability in the results from the recording sectors, accurately ensuring the repeatability of the measurements on clearly defined sectors. Thus, 61 horizontal scan lines (1024 A-scans/line), parallel to the central reference line, were recorded. The scan area used 64 sectors, in the form of an 8 × 8 (3° × 3°) cube, but only the central 2 × 2 and 4 × 4 grids were used and analyzed in our study. The 16 sectors were numbered as in Figure 3, using their divisions into temporal, nasal, superior, and inferior, depending on the position relative to the fovea. The values in micrometers of each sector of the 4 and 16 central macular sectors were recorded and analyzed at the level of: the total macular retinal thickness, inner and outer retinal thickness, and thickness of each individual retinal layer, as mentioned above.

### 2.5. Statistical Analysis

The data collection and graphical representations were performed in Microsoft Excel 2019. For the statistical analysis, IBM SPSS v24 was used. The normality was verified using the Shapiro–Wilk test and, according to its values for the group comparations, we used means and standard deviations. The Student *t* test was performed for the two groups’ comparisons, together with the Levene test for the homogeneity of the variances. For multiple sample comparisons, ANOVA testing was performed, and for a post hoc analysis, a Bonferroni correction was used. The mean difference was considered significant at the 0.05 level.

## 3. Results

A total of 158 eyes were evaluated as follows: 64 eyes of RRMS-NON patients, 26 eyes of RRMS-ON patients, 8 eyes of CIS-NON patients, 6 eyes of CIS-ON patients, and 54 eyes of healthy controls. The age and sex distributions are documented in Table 1.

### 3.1. Peripapillary RNFL Changes in MS

**Figure 4:**
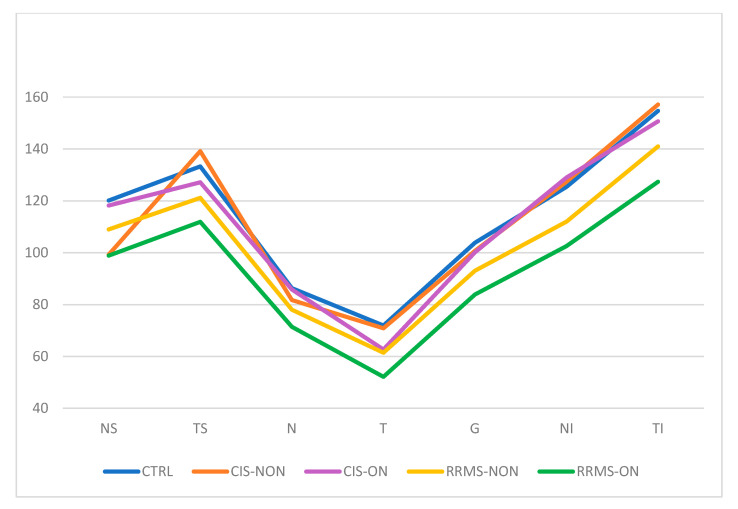
OCT thickness (µm) variation on each pRNFL sector of each group. CTRL—healthy controls; CIS-NON—clinically isolated syndrome without ON history; CIS-ON—clinically isolated syndrome with optic neuritis (ON); RRMS-NON—relapsing remitting multiple sclerosis without ON history; and RRMS-ON—relapsing remitting multiple sclerosis with history of optic neuritis. G—global average, TS—temporal superior, T—temporal, TI—temporal inferior, NI—nasal inferior, N—nasal, NS—nasal superior; and pRNFL—peripapillary nerve fiber layer.

Table 2 summarizes the average thickness of the peripapillary nerve fiber layer (pRNFL) on each individual sector for each group of participants. Figure 4 serves as a visual representation of the pRNFL thickness variation.

Among the RRMS-NON patients, a statistically significant thinning of the pRNFL was observed in all the quadrants compared to the control group (*p* < 0.05). Among the CIS-NON patients, no significant statistical differences were identified in the pRNFL analysis compared to the control group (Table 3), as was the case for RRMS-NON eyes compared to CIS-NON eyes.

Comparing the RRMS-ON and CIS-ON eyes, we observed a statistically significant difference between the pRNFL thicknesses in five of the seven measured sectors: NS (*p* = 0.037), N (*p* = 0.01), G (*p* = 0.006), NI (*p* = 0.013), and TI (*p* = 0.045).

Among the CIS patients, the pRNFL analysis showed that there were no statistically significant differences between those with or without a history of optic neuritis.

Comparing the RRMS patient groups (with and without optic neuritis), a statistically significant difference in the pRNFL thinning in the RRMS-ON patients was noted in the following sectors: G (*p* = 0.002), T (*p* = 0.004), TI (*p* = 0.013), and N (*p* = 0.31).

### 3.2. Macular Thickness Changes in MS

**Figure 5:**
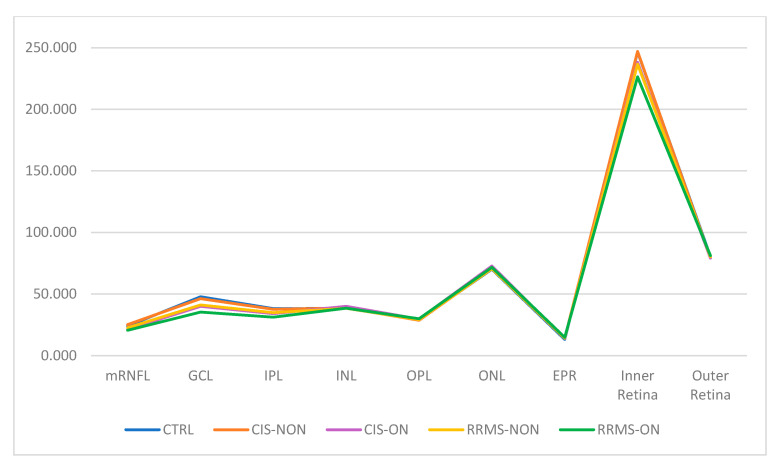
Distribution of the mean values (µm) on the 4 × 4 central macular grid (16 sectors) of each retinal layer.

**Figure 6:**
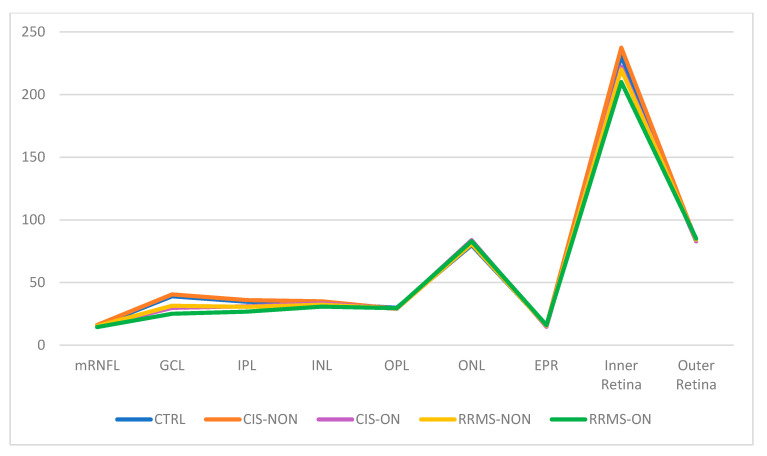
Distribution of the mean values (µm) on the 2 × 2 OCT central macular grid of each retinal layer.

A statistical analysis of the values of the central macular grid of the 2 × 2 and 4 × 4 sectors was performed on each macular layer separately and on the results that provided global indicators such as inner retina, outer retina, and total macular thickness (Table 4 and Table 5, Figure 5 and Figure 6).

Based on our results, in the macular analysis of the NON group for the central 16 sectors grid, we noticed a significant thinning of the mRNFL (*p* = 0.019), GCL (*p* = 0.019), and IPL (*p* < 0.001) in the RRMS eyes compared to the CTRL eyes and also a thinning of both the mRNFL (*p* = 0.007) and GCL (*p* < 0.001) in the RRMS eyes compared to the CIS eyes. For the four central sectors grid, there was a significant decrease in the thicknesses of the GCL and IPL layers between both the RRMS and CTRL groups (*p* = 0.019/*p* = 0.001) and RRMS versus CIS group (*p* < 0.001). No other retinal layers showed significant differences between any groups.

Regarding the ON group (RRMS-ON and CIS-ON), no statistically significant difference could be observed for any of the retinal layers in the central 16 sectors grid analysis. For the four central sectors grid analysis, the study revealed a greater thinning in the RRMS-ON eyes for the following layers: IPL (*p* = 0.019) and INL (*p* = 0.045). However, due to the small number of CIS-ON patients (six), no statistical significance could be highlighted.

An analysis of the cumulative INNER and OUTER retinal layers revealed differences both in the patients without ON and those with previous ON. Among the NON group, there was a significant thinning of the four sectors central grid in the INNER retina thickness in the RRMS eyes compared to both the CTRL (*p* < 0.001) and CIS (*p* = 0.03) eyes. For the 16 sectors central grid, the INNER retina was thinner in the RRMS-NON eyes only when compared to the CTRL group (*p* = <0.001). Analyzing the outer retina thickness in the NON group (CTRL, RRMS-NON, and CIS-NON), the results revealed the lack of a statistically significant difference, for both the 4 and 16 sectors central grids.

Among the ON group, analyzing the inner retina thickness values, we observed a thinning in the RRMS-ON group compared to the CIS-ON (*p* = 0.004) group, only for the central four sectors central grid. Analyzing the OUTER retina thickness for the same group, we noticed an increase in the thickness in the RRMS-ON eyes for both the 4 (*p* = 0.010) and 16 (*p* = 0.031) sectors central grids compared to the CIS-ON eyes.

**Figure 7:**
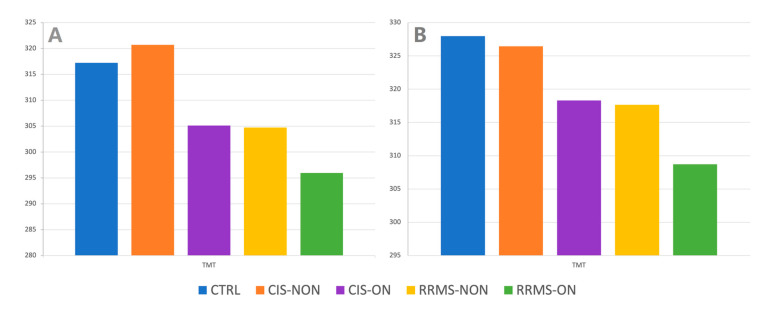
Total macular thickness (TMT) variation on 4 × 4 (**A**), and 2 × 2 (**B**) central macular grid on each group.

Analyzing the TMT for the four sectors central grid in the NON-group, the results showed a thinning of the TMT in the RRMS-NON group compared to both the CTRL (*p* < 0.001) and CIS-NON (*p* = 0.012) groups. For the 16 sectors central grid, the difference was statistically significant only between the RRMS-NON and CTRL groups (*p* = 0.001) (Figure 7).

Regarding the TMT difference between the RRMS-ON and CIS-ON groups, there was no statistically significant value for any of the studied central macular grids (4 and 16 sectors).

Upon comparing the RRMS-ON and RRMS-NON eyes, in the central 16 sectors grid analysis, we found a greater thinning in the ON eyes for the following layers: mRNFL (*p* = 0.001), GCL (*p* < 0.001), IPL (*p* = 0.001), INNER retina (*p* = 0.004), and TMT (*p* = 0.012), and also a thickening of the OUTER retina (*p* = 0.033). For the four central sectors grid, the thinning was significant in the RRMS-ON eyes in the mRNFL (*p* = 0.009), GCL (*p* < 0.001), IPL (*p* = 0.001), INL (*p* = 0.044), inner retina (*p* = 0.003), and TMT (*p* = 0.014).

Comparing the macular thickness for the central 16 sectors between the CIS-ON and CIS-NON groups, there was a thinning of the mRNFL (*p* = 0.002), GCL (*p* = 0.016), and IPL (*p* = 0.035) in the ON eyes. For the four central sectors, the CIS-ON eyes presented a thinning in the mRNFL(*p* = 0.044), GCL (*p* = 0.002), IPL (*p* = 0.013), inner retina (*p* = 0.004), and TMT (*p* = 0.010) compared to the CIS-NON eyes.

## 4. Discussion

Today, SD-OCT allows for in vivo quantifications of axonal loss (pRNFL measurements) and neuronal damage (macular thickness assessments) in MS. Even though it has not yet been included in the multiple sclerosis diagnostic criteria, OCT is recommended as a useful diagnostic and monitoring tool for measuring the neurodegeneration in MS [15].

Our study aimed to assess the retinal differences in two concentric macular grids of the posterior pole, analyzing each retinal layer, along with the optic nerve changes in RRMS and CIS patients (with or without prior optic neuritis) in comparison to healthy controls.

The retinal macular area has the highest density of ganglion cells; therefore, OCT analyses of the macula can be an adequate method of quantifying neuronal damage [17]. Additionally, approximately 50% of the ganglion cells are located in the area 4.5 mm from the center of the fovea (approximately 16 degrees) [18]. The 4 × 4 macular grid analysis approximately covered this area, while the 2 × 2 covered it only partially.

Anatomically, the GCL, mRNFL, and pRNFL constitute the first units within the visual pathway. Irreversible axonal damage at any level can cause transsynaptic retrograde axonal degeneration, which will lead to an atrophy of the inner retinal structures (RNFL and GCIPL) [15].

Optic neuritis is one of the most frequent manifestations of MS. After an acute episode of optic neuritis, the pRNFL has no immediate change and is substantially decreased only at three months [19]. In this study, we only included patients more than 3 months after an ON event. It seems that pRNFL atrophy ends about 6 months after the onset of optic neuritis, the majority of the loss occurring in the first 3 months [20].

GCIPL analyses are reported to be superior compared to pRNFL measurements in tracking the early atrophy caused by optic neuritis, being less influenced by the axonal swelling following edema and inflammation [15,21]. Additionally, GCIPL atrophy after optic neuritis can be detected earlier than pRNFL atrophy, at 1 month after onset [15]. Moreover, if the optic neuritis episode is extremely severe, the pRNFL will show a floor effect, and only the GCIPL will still be useful as a biomarker of visual pathway neurodegeneration [15].

Consistent with previously reported data [15], our study observed significant pRNFL thinning in the RRMS eyes compared to the control group eyes, with the pRNFL atrophy being more severe in the RRMS-ON eyes than the RRMS-NON eyes.

The normal average thickness of the global pRNFL has been shown to be approximately 100 µm. Following an episode of optic neuritis in MS patients, SD-OCT measurements have confirmed previously published data, recording a pRNFL atrophy of 20.10 µm [15]. Our study results regarding the average thinning of the global pRNFL in RRMS-ON patients recorded a similar value of 19.95. The average global pRNFL thinning in the RRMS-NON eyes was 10.79 µm compared to 7.41 µm reported by Petzold et al. [15].

It is already known that ON in MS has an impact on pRNFL thinning, especially in the temporal sector, where papillomacular bundle nerve fibers appear that are important for central vision [22].

We noticed that the RNFL atrophy in the RRMS eyes (both ON and NON eyes) affected all the pRNFL sectors. There was pronounced pRNFL thinning in the RRMS-ON eyes compared to the RRMS-NON eyes identified in the T, TI, and N sectors.

In the presence of optic neuritis, the RRMS eyes suffered a greater decrease in their pRNFL thickness compared to the CIS eyes. This was observed only for the NS, N, NI, and TI sectors.

We could not identify any change in the pRNFL thickness in the CIS-NON eyes when compared to the control group, except for a decrease in thickness observed in the SN sector, which was probably due to the small number of enrolled CIS-NON patients, presenting an unidentified feature. No statistical difference was noticed in the pRNFL thickness between the CIS-ON and CIS-NON eyes. Our results were consistent with previously reported data [23].

We observed no statistically significant difference between he CIS-ON and CTRL eyes regarding the pRNFL, contrary to what Rzepisnki et al. [23] reported. In our study, due to the small sample of CIS-ON patients, even if there was thinning in the pRNFL, especially in the T sector, it could not have gained statistical significance.

We can also conclude that the pRNFL thinning was significantly influenced by the presence of prior optic neuritis in the RRMS patients, emphasizing its value in proving dissemination in the space of characteristic lesions in the CIS-ON patients, as Rzepinski et al. [23] already stated.

Over time, several studies have shown statistically significant decreases in the pRNFL and GCL thicknesses among RRMS patients, regardless of optic neuritis, compared control groups [24,25,26,27].

In their meta-analysis, Petzold et al. [15] reported the atrophy of the GCIPL in both MS-ON and MS-NON eyes compared to controls, the atrophy being more significant in the MS-ON eyes. They also found no changes regarding the ONPL thickness in either MS-ON or MS-NON eyes compared to controls and a minimal increase in its thickness in MSON eyes versus MSNON eyes.

In their study, Martucci et al. [28] created a map of regions of interest in a posterior pole analysis, characterized by statistically significant thinning in MS-NON eyes versus control eyes, with each individual retinal layer showing an irregular damage distribution of the same layers in MS patients. Additionally, in their study, the GCL and IPL damage was more concentrated in the parafoveal area, so we consider it appropriate to analyze the center of posterior pole assessment (comprising our 4 × 4 macular grid).

In our study, the OCT analysis using a 16-sector macular grid of the retinal layers in RRMS-NON eyes revealed a significant thinning for the mRNFL, GCL, and IPL layers compared those in the CTRL group, but only for the mRNFL and GCL when compared to the CIS-NON eyes. Additionally, in this case, the four central sectors of the PP analysis on the RRMS-NON eyes showed atrophy in both the GCL and IPL when compared to either the CTRL or CIS-NON eyes. The macular 2 × 2 mRNFL presented no changes, as its thickness was anatomically decreased in the foveal region.

The changes in the macular area of the RRMS-NON and CIS-NON eyes interest the inner retina layers, regarding retinal ganglion cell layer complex (which is the thickest in macular area [15]). When measuring the TMT and inner retina thickness, there was a significant atrophy in the 2 × 2 and 4 × 4 central grids of the RRMS-NON eyes compared to the CTRL eyes. Contrary to our results regarding TMT, a recent study [28] reported no statistically significant difference in the average retinal thicknesses between RRMS-NON and control eyes when comparing the entire posterior pole grid (64 sectors) analysis, suggesting that macular 2 × 2 and 4 × 4 sector analyses could be more useful for detecting early MS retinal changes. When comparing the RRMS-NON eyes with the CIS-NON eyes, the average values for the TMT and inner retina had a significant decrease only on the 2 × 2 macular grid. There was no significant alteration in the outer retinas in either the RRMS-NON or CIS-NON eyes when comparing them to each other or to the controls. With respect to the controls, the same finding, regarding the non-alteration in the outer retinas in the MS-NON patients, was ascribable to a sparing of the outer retina from neurodegeneration in the absence of ON [29,30,31]. Contrary to our findings, Saidha et al. [32] hypothesized the existence of “primary retinal pathology” as a process totally independent of optic nerve damage, based on their results regarding inner and outer retina thinning being mainly in progressive MS-NON eyes.

In other words, we could say that the closer the macular analysis was performed to the foveal area, the greater the discriminating power of the RRMS-NON changes compared to the CIS-NON eyes. A question arises from this: do the retinal changes in MS start in the central macular area?

In the ON group, the macular analysis showed statistically significant changes in the RRMS-ON eyes when compared only to the CIS-ON eyes, with regard to the decrease in the IPL and INL thickness on the central 2 × 2 macular grid. No changes appeared in the TMT values on either the 2 × 2 or 4 × 4 macular grid. The inner retina was thinner on the 2 × 2 macular grid of the RRMS-ON patients. Additionally, the outer retina displayed an increase in its thickness on both the 2 × 2 and 4 × 4 macular grids in the RRMS-ON eyes compared to the CIS-ON eyes. Although there have been reports of inner and outer retinal alterations in MS patients, best-corrected vision acuity seems to be mainly influenced by inner retinal changes in the parafoveal area [29].

The RRMS-ON eyes recorded a significant thinning of the mRNFL, GCL, IPL, inner retina, and TMT on both the 2 × 2 and 4 × 4 macular grids compared to the RRMS-NON eyes. They also had a decrease in the INL thickness in the 2 × 2 macular area and an increase in the outer retina for the 4 × 4 macular grid. Thus, based on our results, the presence of optic neuritis seems to produce changes regarding all the retinal layers.

The CIS-ON eyes experienced a significant decrease in their ganglion complex cell layers for both the 2 × 2 and 4 × 4 macular grids and a decrease in the inner retina and TMT for only the 2 × 2 macular grid when comparing them to the CIS-NON eyes.

In their study, Eslami et al. [33] and Rzepinski et al. [23] could not find significant differences in the TMV (total macular volume) in either CIS-ON or CIS-NON eyes when comparing them to healthy controls.

Regardless of a prior optic neuritis episode, macular retinal layer changes in MS predominantly affect the ganglion cell complex of the inner retina (consistent with previously published data [15]).

In our study, the OCT analysis of the macular retinal layers in RRMS patients revealed atrophy changes only in the retinal ganglion cell layer complex (mRNFL, GCL, and IPL), which is the thickest in the macular area, with no thinning of the other retinal layers. Our results were consistent with previously published data [15,34]. However, the atrophy of INL could occur in some MS eyes of patients with a longstanding or progressive disease [35]. Significant correlations were also described between the increase in the INL thickness and the decrease in the RNFL/GCIPL volume. The first mentioned appears as a compensatory mechanism for the changes in the other retinal layers [36].

There are several limitations of this study. First of all, we had a relatively small number of participants, mostly in the CIS group.

Secondly, we used only the central 4 and 16 sectors of the macular area, avoiding any interference with large superior and inferior temporal retinal vessels and focusing our analyses on the macular area, where the greatest density of ganglion cells is. Additionally, we used the macular area changes as a surrogate for the global retinal neurodegeneration in MS, as SD-OCT measurements could not analyze the nasal retina. Finally, the OCT data were collected manually.

## 5. Conclusions

Posterior pole analyses could be an important assessment tool for monitoring the protocol of neurodegeneration in multiple sclerosis, thus helping to differentiate MS patients from CIS patients, in the absence of optic neuritis, when using a central 2 × 2 sectors macular grid.

Moreover, the fact that the macula is more affected in MS is an argument that the identified changes are specific to MS and not only related to ON.

The neurodegenerative process affects both the inner retina and pRNFL, with clinical damage appearing for the latter in the following order: CIS-NON, CIS-ON, RRMS-NON, and RRMS-ON. In the presence of optic neuritis, SMRR patients could present alterations in their outer retina too.

## Figures and Tables

**Table 1:** Age and sex distribution.

		CTRL	CIS-NON	CIS-ON	RRMS-NON	RRMS-ON
Sex	F	42	7	5	47	21
	M	12	1	1	17	5
	Mean Age	31.85	30.88	33.83	37.64	30.35

**Table 2:** Descriptive statistics of group analysis of pRNFL thickness (µm) on each sector.

	CTRL		CIS-NON		CIS-ON		RRMS-NON		RRMS-ON	
	Mean	SD	Mean	SD	Mean	SD	Mean	SD	Mean	SD
NS	120.11	24.96	99.25	24.11	118.17	24.11	109.00	23.20	98.88	18.47
TS	133.30	18.66	139.13	17.75	127.17	17.75	121.19	21.95	111.92	26.94
N	86.35	12.88	81.75	7.36	85.83	7.36	78.02	13.18	71.42	12.31
T	71.93	9.63	70.88	10.93	62.67	10.93	61.45	13.39	52.15	13.73
G	103.83	8.62	100.75	9.09	100.17	9.09	93.05	12.39	83.88	12.60
NI	125.39	18.49	127.38	16.15	129.00	16.15	111.98	25.29	102.54	23.17
TI	154.70	20.07	157.13	22.78	150.67	22.78	141.00	22.38	127.35	24.97

CTRL—healthy controls; CIS-NON—clinically isolated syndrome without ON history; CIS-ON—clinically isolated syndrome with optic neuritis (ON); RRMS-NON—relapsing remitting multiple sclerosis without ON history; and RRMS-ON—relapsing remitting multiple sclerosis with history of optic neuritis. G—global average, TS—temporal superior, T—temporal, TI—temporal inferior, NI—nasal inferior, N—nasal, NS—nasal superior; and pRNFL—peripapillary nerve fiber layer.

**Table 3:** The variation in pRNFL thickness (µm) on each sector compared to healthy controls group.

	NS	TS	N	G	T	NI	TI
CIS-NON	−20.86	5.83	−4.60	−3.08	−1.05	1.99	2.42
CIS-ON	−1.94	−6.13	−0.52	−3.67	−9.26	3.61	−4.04
RRMS-NON	−11.11 *	−12.11 *	−8.34 *	−10.79 *	−10.47 *	−13.40 *	−13.70 *
RRMS-ON	−21.23 *	−21.37 *	−14.93 *	−19.95 *	−19.77 *	−22.85 *	−27.36 *

* significant statistical differences, *p* < 0.05; CIS-NON, clinically isolated syndrome without ON history; CIS-ON, clinically isolated syndrome with optic neuritis (ON); RRMS-NON, relapsing remitting multiple sclerosis without ON history; and RRMS-ON, relapsing remitting multiple sclerosis with history of optic neuritis.

**Table 4:** Descriptive statistics of the distribution of the mean values (µm) on the 4 × 4 central macular grid (16 sectors) of each retinal layer.

	CTRL		CIS-NON		CIS-ON		RRMS-NON		RRMS-ON	
	4 × 4 Sectors		4 × 4 Sectors		4 × 4 Sectors		4 × 4 Sectors		4 × 4 Sectors	
	Mean	SD	Mean	SD	Mean	SD	Mean	SD	Mean	SD
mRNFL	24.027	2.343	25.164	1.538	21.427	1.971	22.633	2.592	20.565	2.145
GCL	47.816	2.578	46.164	3.192	40.000	5.085	41.142	6.205	35.315	6.358
IPL	38.182	2.251	37.657	2.522	34.073	3.143	34.919	4.949	31.185	3.584
INL	38.371	2.761	38.711	2.726	39.990	1.931	38.403	2.156	38.505	2.936
OPL	29.113	2.068	28.617	1.926	29.698	4.017	29.216	2.281	29.890	2.492
ONL	70.149	6.128	70.602	6.211	72.709	5.170	70.422	6.971	71.608	6.795
EPR	13.948	1.102	14.047	1.095	13.927	0.749	14.765	1.792	13.927	0.749
Inner Retina	246.603	11.584	247.016	8.100	247.016	8.100	226.382	14.068	238.188	10.118
Outer Retina	80.408	6.183	79.438	1.923	79.438	1.923	81.253	1.674	79.219	3.083
TMT	327.959	12.124	326.430	7.608	326.430	7.608	308.730	12.978	318.302	11.275

CIS-ON, clinically isolated syndrome with prior optic neuritis (ON); RRMS-ON, relapsing remitting multiple sclerosis with prior optic neuritis; CTRL, control group; CIS-NON, clinically isolated syndrome without ON history; and RRMS-NON, relapsing remitting multiple sclerosis without ON history. mRNFL = macular nerve fiber layer; GCL = ganglion cell layer; IPL = inner plexiform layer; INL = inner nuclear layer; OPL = outer plexiform layer; ONL = outer nuclear layer; RPE = retinal pigment epithelium; and TMT = total macular thickness.

**Table 5:** Descriptive statistics of the distribution of the mean values (µm) on the 2 × 2 OCT central macular grid of each retinal layer.

	CTRL		CIS-NON		CIS-ON		RRMS-NON		RRMS-ON	
	2 × 2 Central Grid		2 × 2 Central Grid		2 × 2 Central Grid		2 × 2 Central Grid		2 × 2 Central Grid	
	Mean	SD	Mean	SD	Mean	SD	Mean	SD	Mean	SD
mRNFL	15.431	1.115	16.094	0.743	15.167	0.785	15.215	0.903	14.462	1.254
GCL	39.000	4.300	40.500	3.703	29.792	6.454	31.492	6.485	25.163	6.355
IPL	34.663	3.422	35.969	2.750	31.042	3.610	30.551	4.039	26.837	3.752
INL	33.306	3.830	35.094	3.119	33.417	1.821	32.361	3.344	30.800	2.896
OPL	29.846	2.947	29.031	2.466	29.000	5.736	29.195	3.655	29.558	3.106
ONL	79.830	7.519	80.750	8.169	83.833	7.627	80.944	8.107	83.029	6.859
EPR	15.838	1.231	15.688	1.534	14.750	0.880	15.586	1.449	15.990	1.419
Inner Retina	230.731	14.363	237.344	6.463	222.167	9.497	219.953	14.056	209.990	13.025
Outer Retina	84.019	2.529	83.375	2.722	82.833	2.910	84.195	3.283	85.087	1.512
TMT	317.234	14.780	305.125	7.325	295.960	11.667	295.960	14.940	295.960	13.994

CIS-ON, clinically isolated syndrome with optic neuritis (ON); RRMS-ON, relapsing remitting multiple sclerosis with history of optic neuritis; CTRL, control group; CIS-NON, clinically isolated syndrome without ON history; and RRMS-NON, relapsing remitting multiple sclerosis without ON history. mRNFL = macular nerve fiber layer; GCL = ganglion cell layer; IPL = inner plexiform layer; INL = inner nuclear layer; OPL = outer plexiform layer; ONL = outer nuclear layer; RPE = retinal pigment epithelium; and TMT = total macular thickness.

## Data Availability

The data presented in this study are available on request from the corresponding author.
